# Regulation of Asymmetrical Cytokinesis by cAMP during Meiosis I in Mouse Oocytes

**DOI:** 10.1371/journal.pone.0029735

**Published:** 2012-01-11

**Authors:** Dawei Chen, Yuanwei Zhang, Qiyi Yi, Yun Huang, Heli Hou, Yingyin Zhang, Qiaomei Hao, Howard J. Cooke, Lei Li, Qingyuan Sun, Qinghua Shi

**Affiliations:** 1 School of Life Sciences, University of Science and Technology of China, Hefei, China; 2 Hefei National Laboratory for Physical Sciences at Microscale, Hefei, China; 3 MRC Human Genetics Unit, Institute of Genetics and Molecular Medicine, Western General Hospital, Edinburgh, United Kingdom; 4 Chinese Academy of Sciences, Beijing, China; University of Turin, Italy

## Abstract

Mammalian oocytes undergo an asymmetrical first meiotic division, extruding half of their chromosomes in a small polar body to preserve maternal resources for embryonic development. To divide asymmetrically, mammalian oocytes relocate chromosomes from the center of the cell to the cortex, but little is known about the underlying mechanisms. Here, we show that upon the elevation of intracellular cAMP level, mouse oocytes produced two daughter cells with similar sizes. This symmetrical cell division could be rescued by the inhibition of PKA, a cAMP-dependent protein kinase. Live cell imaging revealed that a symmetrically localized cleavage furrow resulted in symmetrical cell division. Detailed analyses demonstrated that symmetrically localized cleavage furrows were caused by the inappropriate central positioning of chromosome clusters at anaphase onset, indicating that chromosome cluster migration was impaired. Notably, high intracellular cAMP reduced myosin II activity, and the microinjection of phospho-myosin II antibody into the oocytes impeded chromosome migration and promoted symmetrical cell division. Our results support the hypothesis that cAMP plays a role in regulating asymmetrical cell division by modulating myosin II activity during mouse oocyte meiosis I, providing a novel insight into the regulation of female gamete formation in mammals.

## Introduction

Asymmetrical cell division generates unequally sized daughter cells that are destined to acquire different fates. This plays important roles in multiple biological processes [1], [2], [3], [4], [5], [6]. In female mammals, after an exquisite process of homologous chromosome pairing and synapsis, immature oocytes are blocked at prophase I of meiosis with a morphologically visible nucleus, called the germinal vesicle (GV). As the oocyte resumes meiosis in response to hormonal stimulation, it undergoes a process of meiotic maturation to complete meiosis I with an extreme form of asymmetrical cell division. This produces the secondary oocyte and the much smaller first polar body [7], [8], [9], [10]. The secondary oocyte is arrested at metaphase II until fertilization or parthenogenetic activation drives meiotic spindle II to rotate 90 degrees to facilitate the extrusion of the second polar body [11]. During both meiosis I and II, cortical migration and asymmetrical positioning of the meiotic spindle is crucial for the asymmetry of the division [10], [12].

During the meiotic maturation process in mouse oocytes, the meiotic spindle assembles around the site where germinal vesicle breakdown (GVBD) takes place. After assembly, the spindle migrates towards the nearest site on the cortex before anaphase onset [13]. During mitosis, the spindle also migrates to the appropriate location before the initiation of anaphase. Dynamic astral microtubules and cytoplasmic dynein, a minus-end-directed motor protein whose asymmetrical activation and localization is regulated by cortical polarity factors, are proposed to play prominent role in mitotic spindle migration [14]. However, astral microtubules cannot play a similar role in directing metaphase I spindle migration in mammalian oocytes because oocytes lack conventional centrosomes and do not exhibit prominent astral microtubules on spindle poles [15]. In fact, several studies have demonstrated that actin is involved in this spindle migration; for example, the metaphase I spindle remains centrally positioned in oocytes treated with actin polymerization inhibitors [12], [16], [17] or in oocytes lacking the actin nucleator formin-2 [17], [18], [19]. It has also been demonstrated that activated myosin helps to propel the microtubule spindle to the cortex by pulling on the cytoplasmic actin network that extends from the spindle poles to the cortex [19], [20], [21], [22], [23], [24]. These recent studies suggested that myosin pulling on an actin filament network is important for spindle positioning and anchoring to the cortex in mammalian oocytes.

When chromosomes come close to the cortex after spindle migration, they induce cortical differentiation and restrict the position of the cleavage furrow, which is generated by the enrichment of actin filaments and the reduction of microvilli [8], [9], [25]. Furrow ingression is initiated shortly after the formation of cleavage furrow. Notably, the pulling force of myosin on the contractile ring is essential for furrow ingression [22], [26], [27], [28]. Therefore, both of the two critical events of meiotic maturation, spindle migration and furrow ingression, are associated with myosin. However, it is not clear how myosin itself is modulated in mouse oocytes during meiotic maturation.

cAMP, a cyclic nucleotide, plays a key role in regulating female gamete maturation in mammals and some invertebrates [29], [30]. Specifically, meiotic resumption of oocytes is triggered by a decrease in intracellular cAMP levels [31], [32], [33]. The spontaneous meiotic resumption of denuded oocytes can be reversibly prevented by incubation with membrane-permeable cAMP analogs, cAMP phosphodiesterase (PDE) inhibitors or adenylate cyclase activators, such as dibutyryl cAMP (dbcAMP), 3-isobutyl-1-methylxanthine (IBMX) and forskolin [34], [35]. For example, it has been shown that intracellular cAMP in oocytes decreases within 2 hours of the removal of IBMX, and that this decrease promotes the commitment to resuming meiosis I [36], [37]. Because of the roles cAMP plays in meiotic arrest, cAMP analogs, PDE inhibitors and adenylate cyclase activators are widely employed to sustain high intracellular cAMP levels in order to inhibit GVBD in oocyte cultures in vitro. However, it remains unclear whether cAMP plays other roles in the meiotic maturation process of mouse oocytes.

In this study, intracellular cAMP was elevated in mouse oocytes using the chemicals mentioned above (i.e., IBMX, dbcAMP and forskolin) to explore the possible roles of cAMP during meiotic maturation. A large proportion of these chemically treated oocytes underwent symmetrical cell divisions and generated two daughter oocytes with similar sizes. Detailed analyses showed that chromosomes were located in the center of the cell, resulting in a symmetrical cleavage furrow, and thus, symmetrical cell division. Live cell imaging revealed that the migration of the chromosome cluster towards the cortex was significantly suppressed in treated oocytes. Notably, the activity of myosin II (a cAMP-PKA pathway downstream molecule) was impaired in the oocytes with elevated cAMP. Oocytes that had been microinjected with an antibody against activated myosin II also mimicked the severely impaired chromosome migration and symmetrical division caused by elevated cAMP levels. These data provide evidence that cAMP plays a role in regulating chromosome migration by modulating the activity of myosin II, affecting the localization of the cleavage furrow, and thus, asymmetrical cell division in meiosis I.

## Results

### Elevated intracellular cAMP gives rise to abnormal cytokinesis in mouse oocytes

**Figure 1:**
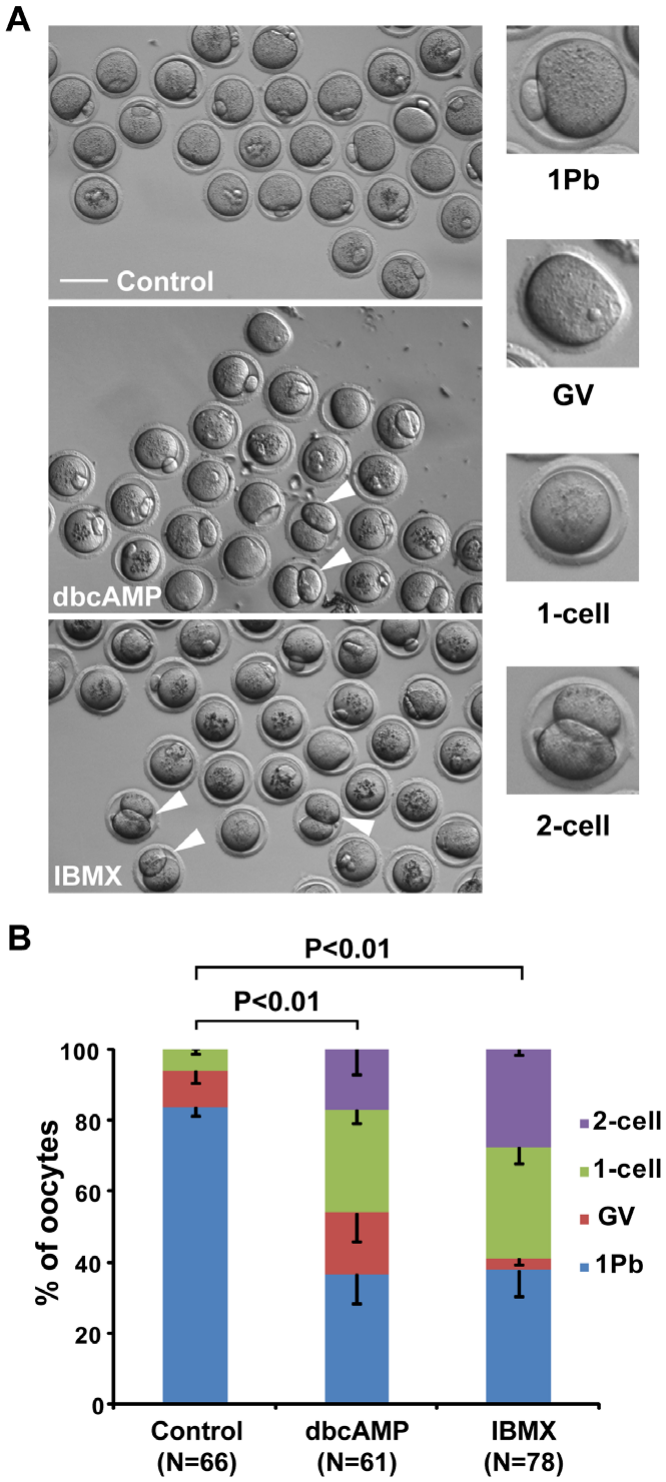
Incubation with dbcAMP or IBMX disrupts asymmetric division in meiosis I in mouse oocytes. Denuded mouse oocytes were cultured in DMSO control medium for 16 hours (control group), or in M16 medium containing 0.3 mM dbcAMP or 0.2 mM IBMX for 24 hours, followed by 16 hours of culture in M16 medium (dbcAMP and IBMX group). (A) Representative images of different oocyte phenotypes after incubation. GV designates oocytes remaining in the GV stage; 1Pb designates mature eggs that extruded a first polar body in the first meiotic division; 1-cell designates oocytes that displayed only one cell; 2-cell designates oocytes that produced two daughter cells of similar sizes in the first meiotic division. Arrowheads are used to indicate 2-cell oocytes. Scale Bar: 100 µm. (B) Comparison of cell division types between oocytes treated with or without IBMX or dbcAMP. Data are the mean ± SEM from 3 independent experiments. *P* values were calculated with a 2×4 χ2-test. N: number of cells analyzed.

IBMX and dbcAMP have been widely used in in vitro culture of oocytes to inhibit GVBD by maintaining high levels of intracellular cAMP [38], [39], [40], [41], [42]. In the present study, oocytes were treated for 24 hours with 0.2 mM IBMX or 0.3 mM dbcAMP. As shown in Figure 1 and movie S1, 83.45±2.28% of oocytes in the control group completed meiosis I by producing a large secondary oocyte and a tiny first polar body (“1Pb” type); 10.51±3.56% remained at the germinal vesicle (GV)-stage (“GV” type); and 6.04±1.36% underwent germinal vesicle breakdown (GVBD) but did not produce daughter cells (“1-cell” type). No oocytes in the control group produced two daughter cells of similar sizes (“2-cell” type) (Fig. 1A). However, in oocytes treated with IBMX or dbcAMP, the percentages of “1Pb” type decreased significantly (37.70±7.22% in IBMX and 36.58±8.46% in dbcAMP groups). The percentage of “GV” type oocytes changed only slightly (4.76±1.63% in IBMX and 17.20±7.89% in dbcAMP groups). Notably, the proportion of “1-cell” type oocytes (31.19±4.52% in IBMX and 29.13±3.84% in dbcAMP groups) and “2-cell” type oocytes (27.86±1.49% in IBMX and 17.09±7.02% in dbcAMP groups) increased significantly in treated groups (Fig. 1A).

**Figure 2:**
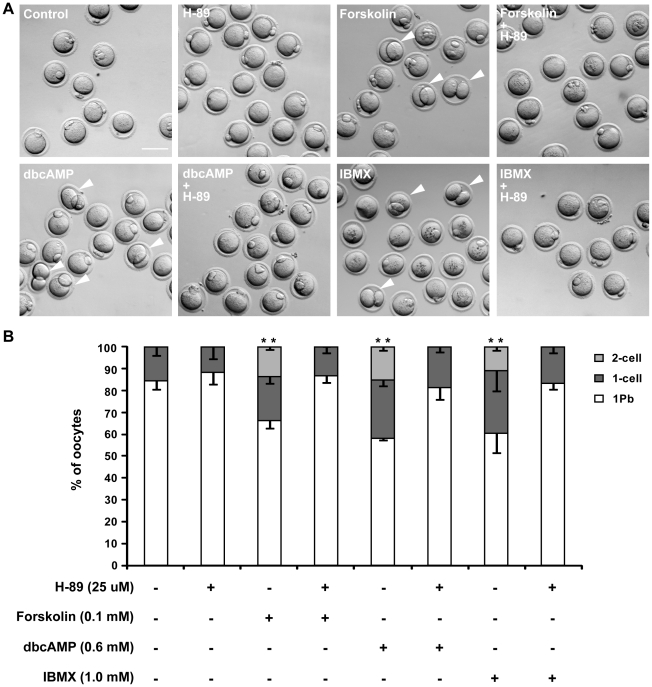
High intracellular cAMP disrupts asymmetric division in post-GVBD oocytes, and H-89 rescues this effect on asymmetric division. Denuded mouse oocytes at the GV stage were collected and cultured in M16 medium for 90 min. Then, oocytes that had undergone GVBD were transferred into DMSO control medium or medium supplemented with different drugs and cultured for 16 hours. (A) Representative images of oocytes after a 16 hour incubation. Arrowheads indicate 2-cell oocytes. Scale bar: 100 µm. (B) Oocyte division results after 16 hours incubation. Data are the mean ±SEM from 3 independent experiments. ** *P*<0.01, 2×3 χ2-test, compared to concurrent control.

The frequency of “2-cell” type oocytes increased in a time-dependent manner after dbcAMP or IBMX treatment (Fig. S1A). To rule out the possibility that these cytokinetic disorders were secondary to delayed GVBD, we performed a set of drug treatment experiments on post-GVBD oocytes. To further confirm that the abnormal cytokinetic results were directly caused by cAMP and its downstream factors, we treated post-GVBD oocytes with a adenylate cyclase activator, forskolin [43], [44], [45], and a PKA inhibitor, H-89 [46], [47], to determine their effects on cytokinesis. As shown in Figure 2, all three cAMP-elevating chemicals promoted both the “2-cell” and “1-cell” type division patterns. We also found that H-89 could rescue the symmetrical division induced by cAMP-elevating chemicals and decrease the frequency of “1-cell” type divisions (Fig. 2B). IBMX/dbcAMP treatment of post-GVBD oocytes also increased the frequencies of both “2-cell” and “1-cell” divisions in a dose-dependent manner (Figs. S1B–C). Altogether, these observations indicate that high intracellular cAMP perturbs the asymmetrical division of primary oocytes.

### Altered cleavage furrow positioning and ingression perturb the asymmetrical division of primary oocytes

**Figure 3:**
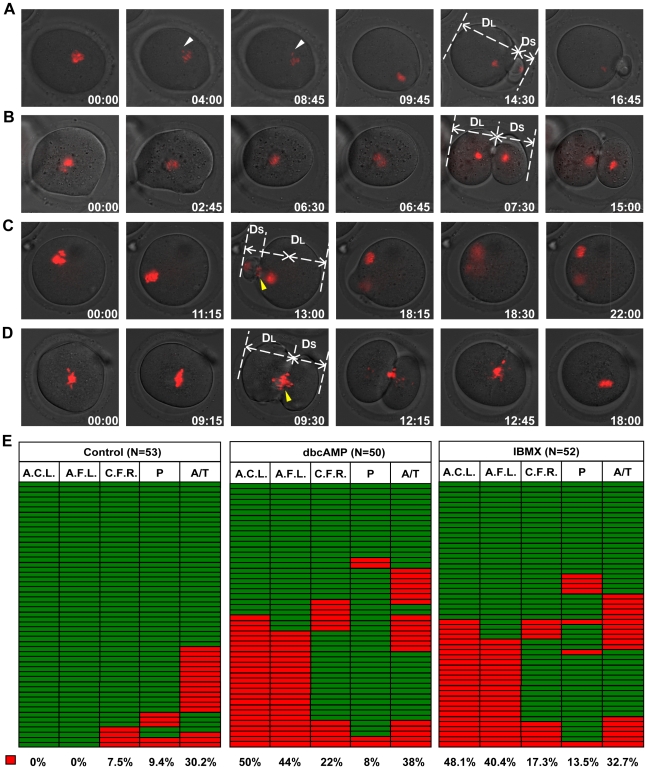
IBMX and dbcAMP treatment perturbed oocyte cytokinesis. Denuded mouse oocytes at the GV stage were collected and cultured in M16 medium for 90 min. Then, oocytes that had undergone GVBD were transferred into DMSO control medium or medium supplemented with 0.6 mM dbcAMP or 1.0 mM IBMX for subsequent live cell imaging. Representative live cell images were collected from oocytes undergoing normal cytokinesis with furrow abscission (A), symmetric cytokinesis with furrow abscission (B), asymmetric cytokinesis with furrow regression (C) and symmetric cytokinesis with furrow regression (D). White arrowheads: prometaphase lagging chromosome. Yellow arrowheads: anaphase/telophase lagging chromosome. The D_L_ and D_S_ in (A), (B), (C) and (D) represent the vertical distances from the far-end cortex to the cleavage furrow in the larger and smaller daughter cells, respectively. Time points (hours∶minutes) indicate the time elapsed from the beginning of imaging. Chromosomes were stained with Hoechst 33342, shown in red. Corresponding movies are provided in the supplemental materials. (E) Analysis of cell division in the control, dbcAMP and IBMX group, with each row representing a single oocyte division. Colour indicates whether the cell showed the presence (red) or absence (green) of abnormal anaphase chromosome localization (A.C.L., first column) or abnormal cleavage furrow localization (A.F.L., second column); occurrence of cleavage furrow regression (red) or not (green) (C.F.R., third column); presence (red) or absence (green) of lagging chromosomes at prometaphase (P, fourth column) or anaphase/telophase (A/T, fifth column). The proportion of red in each column is included below. Chromosome localization was deemed “abnormal” if the ratio of the shortest chromosome-cortex distance at anaphase initiation (D_A_) over that at movie initiation (D_O_) was greater than 0.68 (D_A_/D_0_ >0.68). Furrow localization was deemed “abnormal” if D_L_/D_S_ was less than 1.70 (D_L_/D_S_ <1.70).

To investigate how increased intracellular cAMP perturbs the asymmetrical division of primary oocytes, we carried out time-lapse imaging experiments on post-GVBD oocytes receiving 0.6 mM dbcAMP or1.0 mM IBMX treatment to track the process of meiosis I, with special attention to the localization of the cleavage furrow. To quantitatively describe the localization of the cleavage furrow, the vertical distance from the far-end cortex to the cleavage furrow was measured in both daughter cells, and the ratio of the longer distance over the shorter distance (D_L_/D_S_) was used as a measure of furrow localization (Fig. 3). For those oocytes formed more than one furrow ( Movie S5), we measured the relevant data of the furrows that regressed later than the others. For the 55 control oocytes examined, the average ratio was 3.08±0.13 with a maximum value of 5.34 and a minimum value of 1.68. Based on these data, cleavage furrow localization was defined “abnormal” if the D_L_/D_S_ ratio was less than 1.68. According to the D_L_/D_S_ ratios, 44% and 40.4% of dbcAMP- and IBMX-treated oocytes displayed abnormally positioned cleavage furrows. Notably, all “2-cell” type divisions (10/50 in dbcAMP and 7/52 in IBMX groups) were attributed to the ingression of a centrally localized cleavage furrow (D_L_/D_S_ <1.1, Fig. 3B, Movie S2). Similar results were obtained in oocytes that had been treated with lower concentrations of the two chemicals (0.3 mM dbcAMP, 0.2 mM IBMX) at the GV stage (Fig. S2). Treatment of post-GVBD oocytes with lower chemical concentrations also produced abnormally localized cleavage furrows— over 10% oocytes displayed more symmetrically localized cleavage furrow (Fig. S3), indicating that cAMP drugs at the relatively lower concentration can also affect the normal division. Taken together, these results demonstrate that dbcAMP and IBMX cause cleavage furrows to position centrally, leading to symmetrical oocyte divisions and the subsequent production of “2-cell” type oocytes.

As shown in Figure 3C–D and Movies S3, S4, the “1-cell” type division was caused by cleavage furrow regression. Treatment of oocytes with dbcAMP or IBMX significantly increased the frequency of furrow regression (22% and 17.3% in the dbcAMP IBMX groups, compared to 7.5% of controls). Notably, the regression took place at similar frequencies in oocytes with normally and abnormally localized cleavage furrows (Fig. 3E, Fig. S3B). Interestingly, we also observed two oocytes, one from the dbcAMP group and the other from IBMX group, that formed two simultaneous cleavage furrows; in these oocytes, the two furrows both regressed sequentially ( Movie S5). Taken together, our data indicate that dbcAMP and IBMX treatments increased the likelihood of cleavage furrow regression.

To investigate possible causes of furrow regression, we monitored chromosome movements in both control and treated groups. Frequencies of lagging chromosomes in treated groups were slightly higher than in the control group (Figure 3E), but the differences were not statistically significant (data not shown). In both control and treated groups, lagging chromosomes occurred more frequently in anaphase/telophase than in prometaphase (Figure 3E). No furrow regression was observed when chromosomes lagged only at prometaphse (Figure 3E); besides, over 75% of furrow regression were concomitant with anaphase/telophase chromosome lagging (3/4, 8/11 and 9/9 in control, dbcAMP and IBMX group) (Figure 3C–E, Movies S3, S4, S5), indicating that lagging chromosomes at anaphase/telophase contributes to furrow regression. Interestingly, in the control group, most oocytes with anaphase/telophase lagging chromosomes were able to complete cytokinesis, while in treated groups, about half of the oocytes with anaphase/telophase lagging chromosomes underwent furrow regression (9/19 in dbcAMP group and 9/17 in IBMX group) (Figure 3E). These results indicate that the treatement of cAMP elevating drugs increased the incidence of the lagging-chromosome-related furrow regression.

### Abnormal cleavage furrow positioning is associated with mislocalized chromosome clusters at anaphase onset

**Figure 4:**
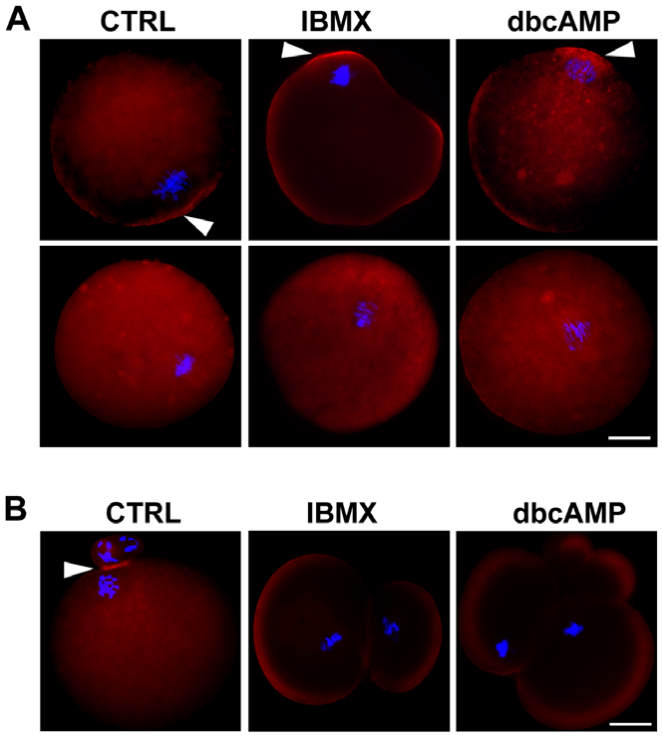
Cortical specification in the primary oocyte is dependent on chromosome cluster position. Representative images of oocytes before cytokinesis initiation (A) and after cytokinesis completion (B). Denuded oocytes were incubated in the presence of 0.3 mM dbcAMP or 0.2 mM IBMX for 24 hours, followed by incubation in M16 for 9 hours, or in control medium in the absence of dbcAMP or IBMX for 10 hours, and subsequently fixed and co-stained with Alexa594-phalloidin (red) and Hoechst 33342 (blue). Oocytes with (row 1 in panel A) or without (row 2 in panel A) an “F-actin cap” were observed in both the control group and treatment groups. Arrowheads indicate “F-actin cap” localization. Scale bar: 20 µm.

Previous studies have highlighted the important role of the chromosome cluster (or meiotic spindle) during polar body formation [8], [9], [12], [48]. Chromosome cluster migration toward the cortex induces cortical differentiation, and thus restricts cleavage furrow position. Cortical differentiation is characterized by actin filament (F-actin) enrichment and microvilli reduction on the cortex [8], [9], [25], [48]. To test whether cortical differentiation was affected by high levels of intracellular cAMP, the F-actin distribution was investigated at the initiation of anaphase or cytokinesis by staining with TRITC-conjugated phalloidin. At the onset of anaphase I, “F-actin caps” were observed in 53.33% and 61.90% of oocytes receiving IBMX and dbcAMP treatment, respectively, but in 82.60% of controls (Fig. 4A). No F-actin cap was observed in oocytes with more centrally located chromosomes, either in the control or treated groups (Fig. 4A, row 2). At the beginning of cytokinesis, an F-actin cap remained visible in all 15 control oocytes, but in only 4/18 IBMX-treated oocytes and 2/11 dbcAMP-treated oocytes. No “F-actin cap” was observed in cells that divided symmetrically after treatment (Fig. 4B). These results not only confirmed that cortical differentiation depends on the location of the chromosome cluster but also indicates that intracellular cAMP may affect chromosome cluster positioning in oocytes.

**Figure 5:**
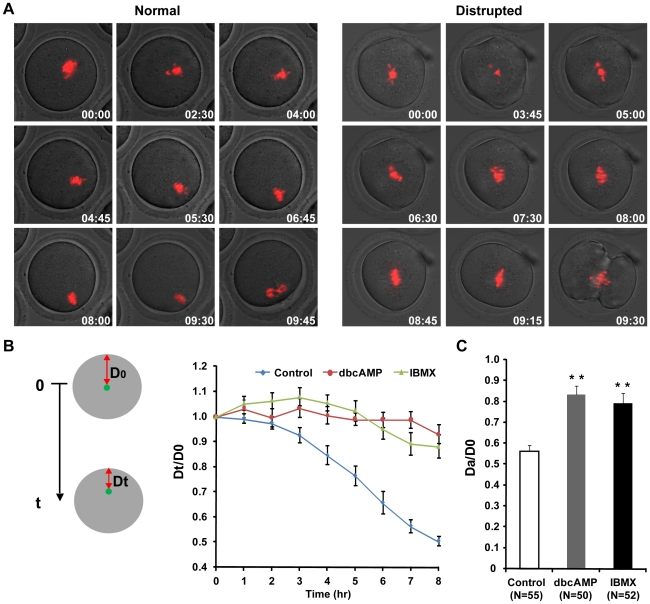
IBMX and dbcAMP treatment of post-GVBD oocytes disturbed chromosome migration. Denuded GV stage oocytes were first cultured in M16 medium for 90 min; after GVBD, the oocytes were transferred into DMSO control medium or medium supplemented with 0.6 mM dbcAMP or 1.0 mM IBMX for live cell imaging. Chromosome movement was tracked during meiosis I. (A) Representative time-lapse images of chromosome movement from imaging initiation to anaphase onset. Time from imaging initiation is shown (hours∶minutes). Red: DNA. (B) Green dots indicate the position of the chromosome cluster. The shortest distance between the chromosomes and the cortex was measured at each time point (Dt) and divided by the distance to the cortex at the beginning of the experiment (D_0_). Dt/D_0_ was used as an indicator of chromosome movement and plotted against time. Panel (C) shows the relative position of the chromosome cluster at anaphase onset, as D_A_/D_0_. D_A_ is the shortest distance between the chromosomes and the cortex at anaphase initiation. ** *P*<0.01, Student's *t*-test, compared to concurrent control. N: number of cells analyzed.

Because cortical differentiation is dependent on the localization of chromosome clusters and restricts the position of the cleavage furrow, it is possible that mislocalization of the cleavage furrow could be caused by abnormal chromosome position in oocytes. To test this hypothesis, we investigated the relationship between chromosome cluster positioning and furrow localization using live cell imaging. To quantify the localization of the chromosome cluster, the shortest distance between the chromosome cluster and the cortex was measured at movie initiation (D_0_) and anaphase onset (D_A_), and the D_A_/D_0_ ratio was used as a measurement of chromosome position at anaphase onset. In the 55 control oocytes, D_A_/D_0_ averaged 0.46±0.01 with a maximum of 0.70 and a minimum of 0.27. Based on the range of D_A_/D_0_ ratios from the control group, we defined chromosome cluster positioning as “abnormal” if the D_A_/D_0_ ratio was greater than 0.70. Abnormally localized chromosome clusters were observed in 44% and 40.4% of oocytes treated with dbcAMP and IBMX, respectively (Fig. 3E). The average ratio of D_A_/D_0_ was significantly greater in the treated oocytes (0.83±0.04 in dbcAMP and 0.79±0.05 in IBMX groups) than in the controls (0.56±0.03) (Fig. 5C). Notably, all cells with abnormally localized cleavage furrows displayed abnormal chromosome cluster positioning at anaphase onset, and no centrally localized cleavage furrows were observed in oocytes with normally positioned chromosome clusters (Fig. 3E). Therefore, our data demonstrate that the formation of abnormally localized cleavage furrows is dependent on the abnormal positioning of chromosome clusters at anaphase onset in mouse oocytes.

### Impeded chromosome migration leads to abnormally positioned chromosome cluster at anaphase onset

To investigate how the abnormal positioning of chromosomes occurred, we utilized live cell imaging techniques to monitor the movement of chromosome clusters during meiosis I. To quantify the movement of the chromosome cluster, the shortest distance from chromosome cluster to the cortex was measured at movie initiation (D_0_) and every hour thereafter (Dt) until the oocytes examined initiated anaphase (Fig. 5B). The ratio Dt/D_0_ was used as a parameter for chromosome cluster localization, and the change of the Dt/D_0_ was used as an indicator of the movement of chromosome clusters during prometaphase I. In the control group, Dt/D_0_ began to decrease at 3 hours after movie initiation and continued to decrease until anaphase onset, when the chromosome clusters were observed to be adjacent to the cortex (Fig. 5A). In contrast, Dt/D_0_ did not change significantly in either IBMX- or dbcAMP-treated oocytes during the first 8 hours after the beginning of imaging (Fig. 5B), and the chromosome clusters remained almost completely still (Fig. 3C, 3D and 5A). At the initiation of anaphase, D_A_/D_0_ was significantly greater in the treated oocytes (0.83±0.04 in dbcAMP and 0.79±0.05 in IBMX) than in the controls (0.56±0.03) (Fig. 5C). The suppression of chromosome migration was also observed in oocytes that had been treated with lower concentrations of the two chemicals (0.3 mM dbcAMP, 0.2 mM IBMX) at the GV stage (Fig. S4) Thus, these data indicate that the abnormal localization of chromosomes at anaphase onset can be attributed to the impaired cortex-ward migration of the chromosome cluster.

### Decreased myosin II activity impedes chromosome cluster migration in oocytes

**Figure 6:**
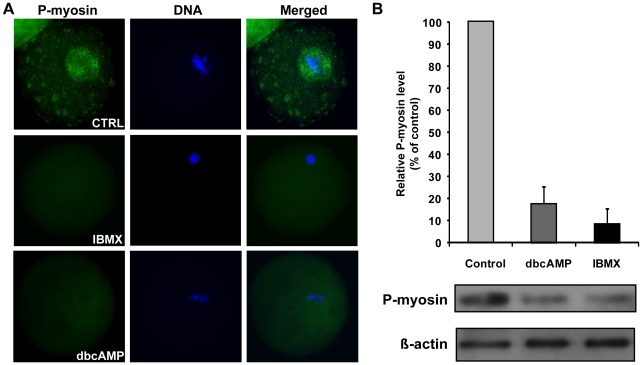
IBMX and dbcAMP treatment decreased myosin II activity in mouse oocytes. (A) Immunostaining of control (CTRL) oocytes cultured in control medium for 10 hours and oocytes cultured in M16 medium for 10 hours after a 20 hour incubation with 0.3 mM dbcAMP or 0.2 mM IBMX. Oocytes were stained with anti-phospho-MLC2 (Ser19) antibody (myosin II, green) and Hoechst 33342 (DNA, blue). (B) The myosin II activities of control or treated oocytes were assayed. Extracts from control, dbcAMP-treated and IBMX-treated oocytes were immunoblotted with antibodies against phospho-MLC2 or β actin. Each lane represents a protein sample derived from 300 oocytes.

It has been reported that activated myosin II is a downstream effector of the cAMP-PKA pathway [49], [50], [51] and plays important roles in the regulation of spindle migration and asymmetrical division in meiosis I [19], [20], [21], [22], [23], [24]. Because myosin II is activated by the phosphorylation of its regulatory light chain (MLC) at Ser19/Thr18 [52], we carried out immunostaining of phospho(Ser19)-myosin II to detect myosin II activity in control, IBMX-treated and dbcAMP-treated oocytes. The results showed that all control oocytes (20/20) displayed strong staining for phosphorylated MLC around the chromosome cluster, whereas only 61.5% (24/39) and 58.8% (10/17) of IBMX- and dbcAMP-treated oocytes did so (Fig. 6A), indicating that myosin II activity around the chromosomes was reduced in IBMX- and dbcAMP-treated oocytes. Immunoblotting against phospho(Ser19)-myosin II presented further evidence that treatment with dbcAMP or IBMX decreased myosin II activity in mouse oocytes (Fig. 6B).

**Figure 7:**
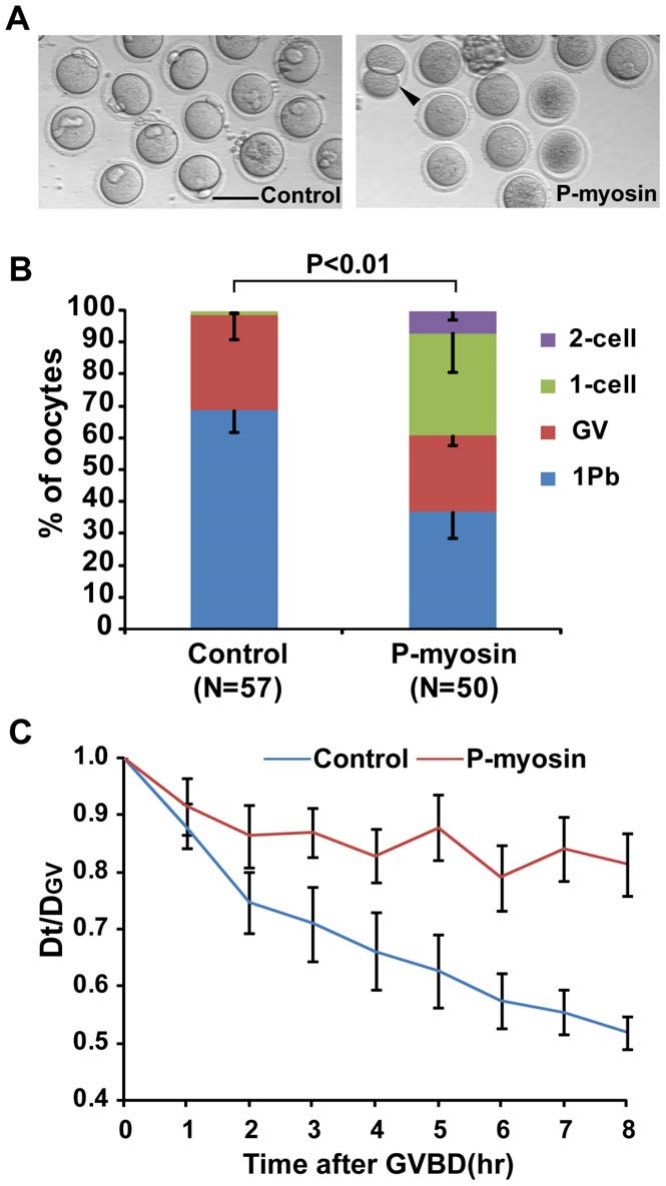
Microinjection of oocytes with monoclonal anti-phospho-myosin antibody facilitated abnormal cytokinesis in meiosis I. (A) Representative images of oocytes taken 16 hours after microinjection with DAM-488 2^nd^ antibody (Control) and anti-phospho-MLC2 (Ser19) antibody (P-myosin). The arrowhead indicates a symmetrically dividing oocyte. Scale bar: 100 µm. (B) The frequency of 1 Pb, GV, 1-cell and 2-cell type divisions in the Control and P-myosin groups. The *P* value was calculated using a 2×4 χ2-test. (C) The shortest distance between chromosomes and the cortex was measured in 15 oocytes per group at GVBD (D_GV_) and hourly time points after GVBD (Dt). Dt/D_GV_ plotted against time was used as an indicator of chromosome movement.

To determine whether decreased myosin activity could affect the asymmetry of cytokinesis, we microinjected phospho-MLC II (Ser19) antibodies into oocytes to inhibit myosin activity. The results showed that 7.17±2.98% of anti-phospho-MLC microinjected oocytes underwent 2-cell type divisions, whereas no 2-cell divisions were observed in control oocytes that were microinjected with donkey anti-mouse IgG (Figs. 7A and B). We also used live cell imaging to follow chromosome movement in these oocytes. In control oocytes, chromosomes migrated towards the cortex beginning shortly after GVBD. In contrast, chromosomes remained centrally located in the anti-phospho-MLC microinjected oocytes (Fig. 7C). Similarly, treatment with blebbistatin, an inhibitor of myosin II [53], generated symmetrical divisions in 17 out of 43 oocytes (Fig. S5). Therefore, the reduced myosin activity in oocytes impaired chromosome migration and thus contributed to the symmetrical cytokinesis of meiosis I.

## Discussion

This study showed that the administration of chemicals that elevate intracellular cAMP caused some oocytes to undergo symmetrical cell division during meiosis I, thus producing two daughter cells with similar sizes. This symmetrical cell division could be rescued by inhibiting PKA, a cAMP-dependent protein kinase. Detailed analyses showed that the impaired migration of chromosome clusters led to the symmetrical localization of cleavage furrows and thus to symmetrical cell division. The activity of myosin II, downstream of the cAMP-PKA pathway, was decreased when intracellular cAMP was elevated and microinjection of oocytes with antibodies against activated myosin II severely impeded chromosome migration toward the cortex during meiosis I and resulted in symmetrical divisions. These results provide evidence that cAMP plays a role in modulating chromosome migration by regulating myosin II activity, subsequently affecting cleavage furrow localization and the asymmetrical division of meiosis I in oocytes.

An interesting and novel discovery in this study is that oocytes with elevated intracellular cAMP do not position the meiotic spindle adjacent to the cortex, which is required to form a polar body. During the maturation process in these oocytes, the centrally formed metaphase I spindle failed to translocate to the cortex, remaining in the center of the oocyte until anaphase onset (Fig. 5, Movie S2). During mitosis, the spindle needs to migrate to the appropriate location before anaphase initiation, a process that is proposed to rely on dynamic astral microtubules and the minus-end-directed MT motor protein dynein [14]. However, astral microtubules are unlikely to play a similar role in directing metaphase I spindle migration in mammalian oocytes, due to the lack of conventional centrosomes and prominent astral microtubules on spindle poles [15]. Studies of the actin cytoskeleton have demonstrated that actin is involved in spindle positioning during meiosis I; the metaphase I spindle remains centrally positioned in oocytes that have been treated with actin polymerization inhibitors [12], [16], [17] or lack the actin polymerization activator formin-2 [17], [18], [19]. However, the actin cytoskeleton appeared normal in oocytes with elevated cAMP, in that all treated oocytes initiated furrow formation (Fig. 3, Fig. S2, Fig. S3), which is known to be driven by the actomyosin-based contractile ring [54], though not all oocytes accomplished abscission of the cleavage furrow (Fig. 3, Fig. S2, Fig. S3). Clearly, increased cAMP did not grossly distort the actin cytoskeleton, although asymmetrical spindle positioning did not occur.

A recent study by Schuh and Ellenberg [24] suggests that myosin pulling on actin contributes to the motion of the meiotic spindle. They propose that activated myosin helps to propel the microtubule spindle to the cortex by pulling on the cytoplasmic actin network that extends from the spindle poles to the cortex. Their model, which has been experimentally verified, predicts that spindles will move end-on to the nearest cortex and that motion will speed up as the spindle nears the cortex. Consistent with this role for myosin, we observed activated, phosphorylated myosin light chain protein concentrated adjacent to the meiotic spindle in untreated oocytes (Fig. 6A). Additionally, microinjection of phospho-myosin II antibody into oocytes reduced the rate of chromosome migration and promoted symmetrical cell division (Fig. 7). Similarly, we observed decreased myosin activity in oocytes with elevated cAMP (Fig. 6). Our study confirms the important role of myosin for spindle positioning and anchoring to the cortex in mammalian oocytes.

In meiosis I mouse oocytes, when chromosomes come close to the cortex after spindle migration during meiosis I, they induce cortical differentiation which restricts the position of the cleavage furrow; this differentiation is reflected by enrichment of actin filaments and reduction of microvilli [8], [9], [10], [25], [55]. Consistently, our results of F-actin immunostaining demonstrated that cortical differentiation occurred when the chromosomes approached the oocyte cortex (Fig. 4). It is also worth noting that no F-actin cap was observed in oocytes with more centrally positioned chromosomes. Live cell imaging analysis revealed that all oocytes were able to initiate cleavage furrow formation, regardless of whether chromosomes were centrally or cortically positioned (Fig. 3, Fig. S2, Fig. S3). This suggests that cortical differentiation has a positive, but nonessential, role in furrow formation. Our observations are inconsistent with the proposal that furrow induction by the spindle midzone in the oocyte is distance-dependent, as suggested by Wang et al. [56]. In that study, centrally formed spindle midzones induced neither membrane furrow nor cytokinesis, while subcortical spindle midzones induced cortical furrowing and polar body extrusion after egg activation [56]. This inconsistency suggests a probable difference in the mechanisms regulating furrow formation in meiosis I and meiosis II in mammalian oocytes.

Most of the furrow regression incidents, which were more frequent in treated oocytes than in control oocytes, were concomitant with anaphase/telophase lagging chromosome (Figs. 1B and 3C–E). One mechanism that could explain this finding is the presence of chromatin in the cleavage furrow, which has been reported to interfere with the completion of cytokinesis [57], [58]. Another interesting discovery is that oocytes with anaphase/telophase lagging chromosomes in treated groups displayed much higher rates of cleavage furrow regression than those oocytes in control group (Figure 3E). Given that the myosin activity was reduced in treated oocytes (Fig. 6) and the pulling force of myosin on the contractile ring is essential for cytokinesis completion [22], [26], [27], [28], [59], it is reasonable to suggest that the higher rates of furrow regression in treated oocytes are attributable to the reduced myosin II activity. Besides, it seems that furrow regression and furrow localization are independent (Fig. 3, Fig. S2, Fig. S3),which is consistent with previous studies [60].

The chemicals used in this study elevate intracellular cAMP by acting as a cAMP mimic (dbcAMP) [61], by preventing the PDE-dependent degradation of cAMP (IBMX) [62], or by overactivating adenylyl cyclase (forskolin). The PKA inhibitor H-89 was able to inhibit the symmetrical division induced by high intracellular cAMP (Fig. 2), indicating that cAMP regulates asymmetrical division during oocyte meiosis I by activating cAMP-dependent PKA. This is inconsistent with work by Wang et al. in Xenopus, which suggested that PKA activity is restrictive only up to GVBD [63]. The later stages of oocyte maturation, including formation of the metaphase I spindle, homolog separation (anaphase), emission of the first polar body and formation of metaphase II spindle, are not sensitive to PKA activation [63]. One likely explanation is the species difference — the cAMP-PKA regulation pathway may not be identical in rodents and Xenopus, and mice may thus be more susceptible to cAMP-induced PKA activation. Newhall et al. proposed in 2006 that type II PKA translocated from the cytosol [64], [65], [66] to mitochondria to ensure GVBD initiation [67]. Given that the knockout mice used by Newhall et al. were not completely infertile [67], and that soluble (cytosolic) type I PKA is present in rodent oocytes [68], we speculate that the cAMP may have activated type I PKA in the post-GVBD oocytes in our study. Increased PKA activity could attenuate RhoA activation and thus reduce Rho-kinase activity, which is responsible for myosin II regulatory light chain activation [69], [70], [71]. Additionally, activated PKA phosphorylates MLCK (Myosin Light Chain Kinase), thereby reducing its activity and leading to decreased myosin activity [72]. Consistent with these theories, we observed reduced myosin activity in drug treated oocytes (Fig. 6). Additionally, specifically suppressed myosin activity in oocytes significantly reduced the chromosome migration rate (Fig. 7). This suggests that as a downstream effector of cAMP-PKA pathway, myosin II is responsible for chromosome migration defects and subsequent abnormalities in cytokinesis, indicating that the cAMP-PKA pathway might be involved in the regulation of asymmetrical chromosome positioning.

In summary, this study indicates that the cAMP-PKA pathway contributes to the regulation of chromosome subcortical location and cytokinesis in mouse oocyte meiosis I. The regulatory role of the cAMP-PKA pathway in chromosome migration and cleavage furrow ingression may be exerted through myosin II. This is the first time that the cAMP-PKA pathway has been reported to regulate not only meiosis resumption but also other meiotic events in mouse oocytes. Moreover, it has recently been proposed that reduced non-muscle myosin activity could cause cytokinesis failure and multipolar mitosis in cultured somatic cells [22]. Therefore, the mitotic and meiotic side effects of chemicals that are widely used to activate the cAMP-PKA pathway for therapeutic or agricultural purposes should be considered carefully prior to their use.

## Materials and Methods

ICR female mice aged 3–6 weeks were purchased from the National Rodent Laboratory Animal Center (Shanghai Branch, China). The collection and using of mice oocytes were under the approval of the Institutional Review Board at University of Science and Technology of China, the approval ID: USTCAU201000004.

### Chemicals

All chemicals used in this study were purchased from Sigma Chemical Company (St. Louis, MO, USA) unless otherwise noted. Stock solutions of IBMX (0.5 M), forskolin (10 mM) and H-89 (10 mg/ml) were dissolved in dimethyl sulfoxide (DMSO), stock solution of dbcAMP (30 mM) were dissolved in distilled water. All the stock solutions were stored at −20°C and diluted with culture medium prior to use. In the cases of treatment on GV-stage oocytes, the control group employed M16 medium with correspondent DMSO concentration. In the cases of treatment on post-GVBD oocytes, the DMSO concentration of the control medium was at the highest level used in the different experimental treatments.

### Collection and culturing of denuded oocytes

Mice were sacrificed by cervical dislocation. Cumulus-oocyte complex (COC) were released from antral follicles by puncturation in M2 medium (Sigma-Aldrich, Cat. No. M7167). After being pipetted repeatedly, cumulus cells were removed from COC. Only denuded oocytes showing clear nuclear membrane (GV oocytes) were collected. Oocytes were washed 3 times in M2 medium then transferred into M16 medium (Sigma-Aldrich, Cat. No. M7292). Oocytes were cultured in M16 medium covered by mineral oil (Sigma-Aldrich, Cat. No. M8410), in a humidified atmosphere of 5% CO2 at 37°C. For post-GVBD treatment, oocytes were first cultured in M16 medium for 90 min, then the oocytes which had underwent GVBD were transferred into M16 medium with IBMX/dbcAMP/forskolin/H-89 for following experiments. Before use, all the media were pre-equilibrated in an incubator with 5% CO_2_ and 100% humidity at 37°C.

### Microinjection of denuded oocytes

Monoclonal antibody of phospho-myosin II light chain (Cell Signaling Techonology, Cat. No. 3675) or donkey anti-mouse antibody conjugated with Alexa Fluor 488 (Molecular Probes, Cat. No. A21202) was diluted at 1∶500 and microinjected into the cytoplasm of GV oocytes with an Eppendorf TransferMan NK2 microinjector and borosilicate glass capillaries (World Orecision Instruments, Cat. No. TW100F-6). The oocytes were maintained in M2 medium during microinjection, then were cultured in M16 medium in a humidified atmosphere with 5% CO_2_ at 37°C.

### Immunostaining

For phospho-myosin II staining, the oocytes were fixed with 3.7% paraformaldehyde in PBS for 1 hour at 30°C, and then rinsed 3 times in blocking solution (1% BSA in PBS) followed by permeabilization with 0.25% Triton X-100 in PBS for 10 minutes at room temperature. Then, rabbit antibody against Ser19-phospho myosin II light chain-2 (Cell Signaling Technology, Cat. No. 3671) was applied at 1∶100 for 1 hour at room temperature. An Alexa Fluor 488 dye conjugated anti-rabbit antibody (Molecular Probes, Cat. No. A21206) was used at 1∶150 as the secondary antibody. Before the incubation in PBS containing 5 µg/ml Hoechst 33342 for 10 minutes, oocytes were rinsed 3 times in blocking solution (10 minutes each) to reduce nonspecific binding. For F-actin staining, the oocytes were fixed for 25 minutes in 4% paraformaldehyde in PBS, and then permeabilized in PBS containing 0.1% Triton X-100 and 0.3% BSA for 10 minutes. After the incubation for 2 hours in PBS containing 0.01% Triton X-100, 0.03% BSA, and 5 U/ml TRITC-phalloidin at room temperature (Sigma-Aldrich, Cat. No. P1951), the oocytes were rinsed 3 times in PBS containing 0.01% Triton X-100 and 0.03% BSA, followed by an incubation in PBS containing 5 µg/ml Hoechst 33342 for 10 minutes before observation.

### Immunoblotting analysis

Control oocytes were collected for lysates at 10 hours after GVBD, so were those which had been incubated with dbcAMP or IBMX for 20 hours. Western blotting was performed as previously described [73]. Lysates were separated by SDS-PAGE and the proteins were then transferred to nitrocellulose membranes (Amersham Biosciences, Cat. No. RPR303D). The membranes were then blocked in TBST (0.5% Tween-20 in TBS) containing 5% BSA for 1 hours, incubated with a rabbit anti-Ser19-phospho myosin II light chain-2 antibody (Cell Signaling Technology, Cat. No. 3671; 1∶1000) or a mouse anti-beta actin antibody (Abcam, Cat. No. Ab52; 1∶1000) overnight at 4°C. Alkaline phosphatase (AP)-conjugated anti-mouse IgG (Promega, Cat. No. S372B; 1∶1000) or anti-rabbit IgG (Promega, Cat. No. S373B; 1∶1000) were used as secondary detection reagents. Phosphorylated myosin II light chain-2 and beta-actin protein levels were evaluated by the detection of activity of alkaline phosphatase using a Lumi-Phos kit (Pierce Biotechnology, KJ1243353).

### Live cell imaging and analysis

For live cell imaging, oocytes were collected and placed in M16 medium supplemented with 5 ng/ml Hoechst 33342 (Molecular Probes) and chemicals in correspondent concentration on gridded coverglass bottom dishes (MatTek, CatNo. P35G-1.5-7-C-grid). The dishes were placed in a 16 cm×10.8 cm×1.8 cm chamber with humidified 5% CO_2_ delivered into it. The chamber was housed in a custom-made 37°C incubator attached with a microscope.

Images at multiple locations on the coverglass were automatically acquired using a Nikon TE2000E inverted microscope equipped with a 20×Nikon Plan Apo objective, a linearly-encoded stage (Proscan, Prior), a Hamamatsu Orca-ER CCD camera and NIS-Elements AR v3.0 software. Fluorescence illumination was provided by a mercury-arc lamp with two neutral density filters (for a total 128-fold reduction in intensity). Fluorescence and differential interference contrast images were acquired every 15 minutes. The oocytes that entered mitosis at least 10 hours before the end of imaging were analyzed for chromosome cluster dynamics. Only the oocytes that entered anaphase with clearly visible chromosome clusters and cleavage furrows were analyzed for anaphase chromosome-cortex distance and furrow positioning.

### Statistics

The number of oocytes used for each experiment is indicated in the figure legends. Unless otherwise specified, data were collected from three independently replicated experiments and presented as the mean ± SEM. Statistical significance was assessed by means of two-tailed Student's *t*-test or 2×4 chi-square test. In all cases, P<0.05 was deemed significant.

## Supporting Information

Figure S1 **Disturbance of meiosis I induced by dbcAMP and IBMX is time- and dose-dependent.** (A) Denuded mouse oocytes were cultured in M16 medium containing 0.3 mM dbcAMP or 0.2 mM IBMX for different lengths of time. The frequency of “2-cell” divisions (oocytes produced two daughter cells of similar sizes) increased with increased duration of dbcAMP or IBMX treatment. (B) and (C) The frequency of 2-cell and 1-cell divisions increased with increasing concentration of IBMX or dbcAMP. Denuded mouse oocytes were cultured in M16 medium until GVBD and then cultured in M16 medium containing different concentrations of IBMX or dbcAMP for 15 hours. Data represent the mean ± SEM from 3 independent experiments.
(TIF)

Figure S2 **IBMX and dbcAMP treatment perturb oocytes cytokinesis.** Denuded mouse oocytes at the GV stage were cultured in M16 medium supplemented with 0.3 mM dbcAMP or 0.2 mM IBMX for 20 hours, then transferred into drug-free medium for live cell imaging. Control oocytes were collected and cultured in DMSO control medium. Representative live cell images were collected from oocytes undergoing normal cytokinesis with furrow abscission (A), symmetric cytokinesis with furrow abscission (B), asymmetric cytokinesis with furrow regression (C) and symmetric cytokinesis with furrow regression (D). The D_L_ and D_S_ in (A), (B), (C) and (D) represent the vertical distances from the far-end cortex to the cleavage furrow in larger and smaller daughter cells, respectively. Time is shown as hours∶minutes after GVBD. Chromosomes were stained with Hoechst 33342 (red). Panel (E) summarizes the normal and abnormal cytokinesis in control, dbcAMP-treated and IBMX-treated groups, with each row representing a single oocyte division. The cell divisions are indicated in different colors: presence (red) or absence (green) of abnormal anaphase chromosome localization (A.C.L., first column) or abnormal cleavage furrow localization (A.F.L., second column); occurrence of cleavage furrow regression (red) or not (green) (C.F.R., third column). The proportion of red in each column is included below. Chromosome localization was deemed “abnormal” if the ratio of the shortest chromosome-cortex distance at anaphase initiation (D_A_) to that at GVBD (D_GV_) was greater than 0.6 (D_A_/D_GV_ >0.6). Furrow localization was deemed “abnormal” if D_L_/D_S_ was less than 1.9 (D_L_/D_S_ <1.9).
(TIF)

Figure S3 **Incubation with 0.2 mM IBMX or 0.3 mM dbcAMP did not significantly affect post-GVBD oocytes.** Denuded mouse oocytes at the GV stage were collected and cultured in M16 medium for 90 min; then, oocytes that had undergone GVBD were transferred into DMSO control medium or medium supplemented with 0.3 mM dbcAMP or 0.2 mM IBMX for live-cell imaging. Shown is a summary of normal and abnormal cytokinesis in control, dbcAMP and IBMX groups, with each row representing a single oocyte division. The cell divisions are indicated in different colors: presence (red) or absence (green) of abnormal anaphase chromosome localization (A.C.L., first column) or abnormal cleavage furrow localization (A.F.L., second column); occurrence of cleavage furrow regression (red) or not (green) (C.F.R., third column). The proportion of red in each column is included below. Chromosome localization was deemed “abnormal” when D_A_/D_O_ >0.68. The vertical distances from the far-end cortex to the cleavage furrow in larger and smaller daughter cells were measured in each oocyte, and furrow localization was deemed “abnormal” if D_L_/D_S_ <1.70.
(TIF)

Figure S4 **IBMX and dbcAMP treatment on oocytes at GV stage disturbed chromosome migration.** Chromosome movement was tracked by live cell imaging in control oocytes, and the oocytes treated for 20 hours with 0.2 mM IBMX or 0.3 mM dbcAMP. (A) Representative time lapse images of chromosome movement from GVBD to anaphase onset. The time of GVBD was set as 00∶00 (hours∶minutes). Red: DNA. (B) Green dots indicate the position of the chromosome cluster. The shortest distance between the chromosomes and the cortex was measured at each time point (Dt) and divided by the distance to the cortex at GVBD (D_GV_). Dt/D_GV_ plotted against time was used as an indicator of chromosome movement. Panel (C) shows the relative position of the chromosome cluster (i.e., average D_A_/D_GV_) at anaphase onset. D_A_ is the shortest distance between the chromosomes and the cortex at anaphase initiation. *P* values are from a *t*-test.
(TIF)

Figure S5 **Blebbistatin induces symmetric meiosis I division in mouse oocytes.** (A) Oocytes were incubated with (Bleb) or without (CTRL) 0.2 mM blebbistatin for 16 hours. Arrowheads indicate oocytes that produced 2 daughter cells of similar size (“2-cells”). (B) The frequency of 2-cells was elevated by the presence of 0.2 mM blebbistatin.
(TIF)

Movie S1 **A representative time-lapse movie showing a mouse oocyte that completes normal meiosis I.** Acquisition time is shown as hours: minutes: seconds in the upper left corner.
(AVI)

Movie S2 **A representative time-lapse movie showing a mouse oocyte that completes symmetric cytokinesis.** Acquisition time is shown as hours: minutes: seconds in the upper left corner.
(AVI)

Movie S3 **A representative time-lapse movie showing a mouse oocyte with asymmetrically positioned cleavage furrow that undergoes furrow regression eventually.** Acquisition time is shown as hours: minutes: seconds in the upper left corner.
(AVI)

Movie S4 **A representative time-lapse movie showing a mouse oocyte with symmetrically positioned cleavage furrow that undergoes furrow regression eventually.** Acquisition time is shown as hours: minutes: seconds in the upper left corner.
(AVI)

Movie S5 **A representative time-lapse movie showing a mouse oocyte with two simultaneous formed cleavage furrows that regressed sequentially.** Acquisition time is shown as hours: minutes: seconds in the upper left corner.
(AVI)
