# Supplementary figures and images for: Regulation of Asymmetrical Cytokinesis by cAMP during Meiosis I in Mouse Oocytes
Source: PLoS One. 2012 Jan 11;7(1):e29735. doi: 10.1371/journal.pone.0029735 (PMC3256179; doi:10.1371/journal.pone.0029735)

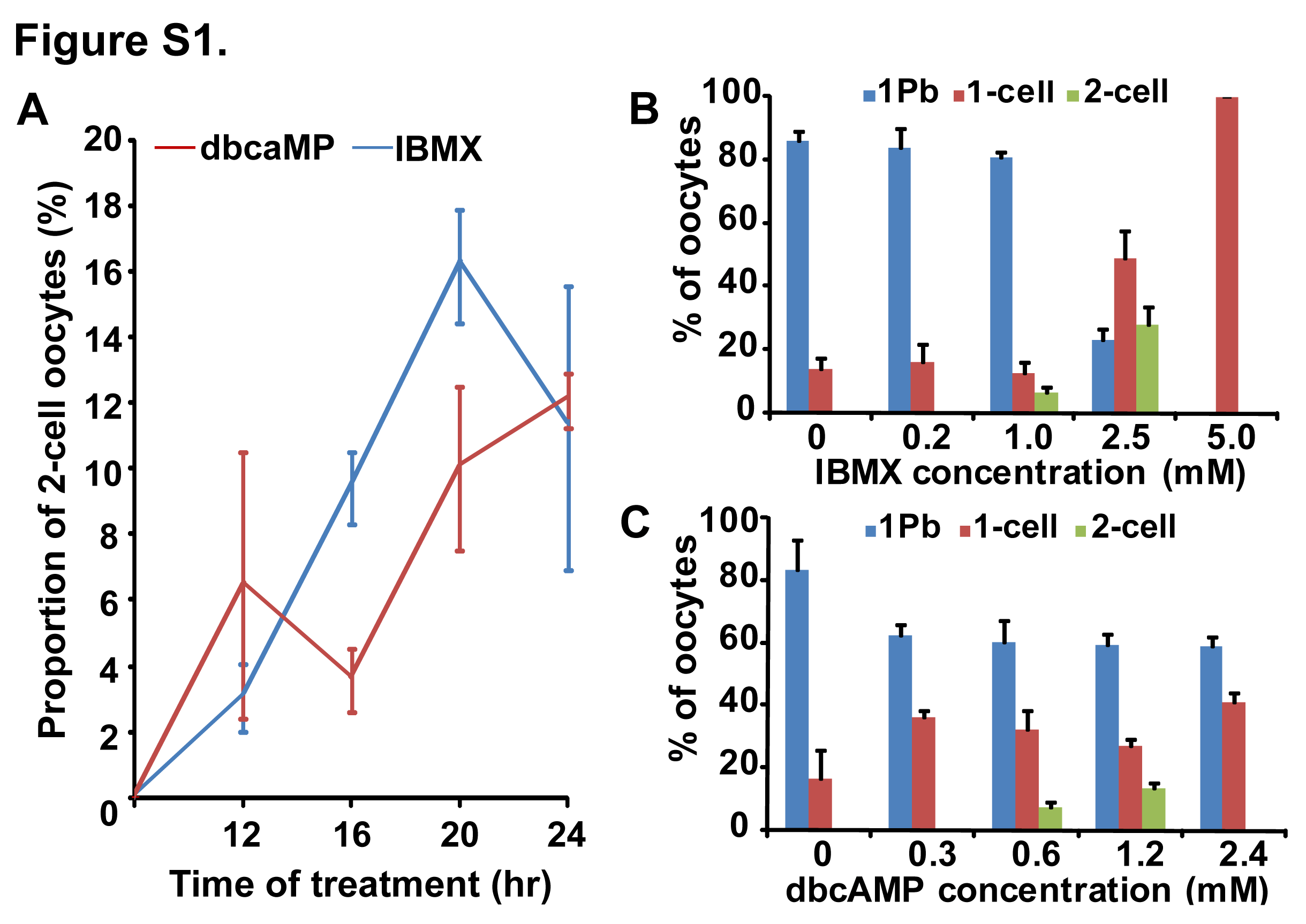

Supplement: Figure S1 — Disturbance of meiosis I induced by dbcAMP and IBMX is time- and dose-dependent. (A) Denuded mouse oocytes were cultured in M16 medium containing 0.3 mM dbcAMP or 0.2 mM IBMX for different lengths of time. The frequency of “2-cell” divisions (oocytes produced two daughter cells of similar sizes) increased with increased duration of dbcAMP or IBMX treatment. (B) and (C) The frequency of 2-cell and 1-cell divisions increased with increasing concentration of IBMX or dbcAMP. Denuded mouse oocytes were cultured in M16 medium until GVBD and then cultured in M16 medium containing different concentrations of IBMX or dbcAMP for 15 hours. Data represent the mean ± SEM from 3 independent experiments. (TIF) [file pone.0029735.s001.tif]

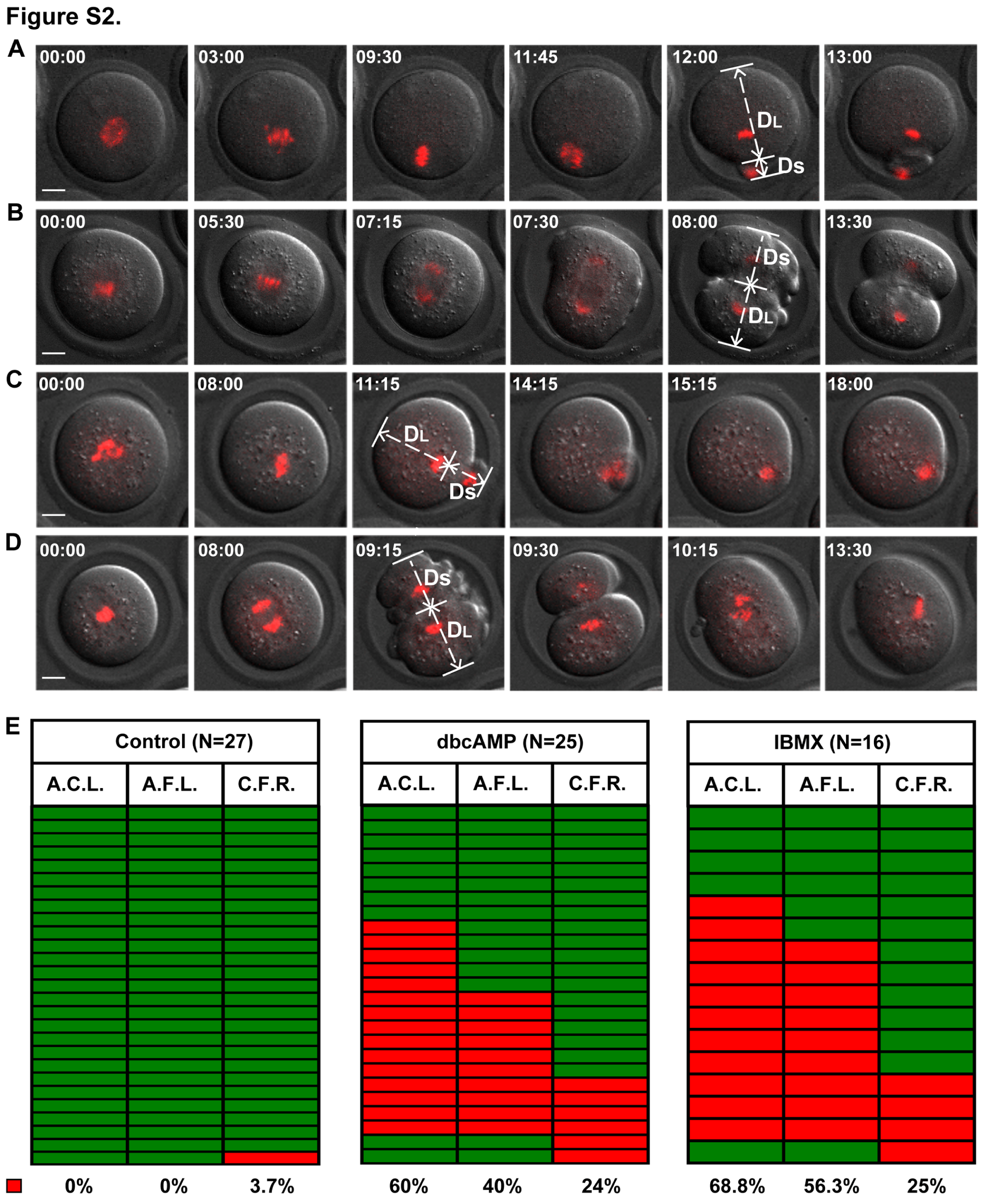

Supplement: Figure S2 — IBMX and dbcAMP treatment perturb oocytes cytokinesis. Denuded mouse oocytes at the GV stage were cultured in M16 medium supplemented with 0.3 mM dbcAMP or 0.2 mM IBMX for 20 hours, then transferred into drug-free medium for live cell imaging. Control oocytes were collected and cultured in DMSO control medium. Representative live cell images were collected from oocytes undergoing normal cytokinesis with furrow abscission (A), symmetric cytokinesis with furrow abscission (B), asymmetric cytokinesis with furrow regression (C) and symmetric cytokinesis with furrow regression (D). The DL and DS in (A), (B), (C) and (D) represent the vertical distances from the far-end cortex to the cleavage furrow in larger and smaller daughter cells, respectively. Time is shown as hours∶minutes after GVBD. Chromosomes were stained with Hoechst 33342 (red). Panel (E) summarizes the normal and abnormal cytokinesis in control, dbcAMP-treated and IBMX-treated groups, with each row representing a single oocyte division. The cell divisions are indicated in different colors: presence (red) or absence (green) of abnormal anaphase chromosome localization (A.C.L., first column) or abnormal cleavage furrow localization (A.F.L., second column); occurrence of cleavage furrow regression (red) or not (green) (C.F.R., third column). The proportion of red in each column is included below. Chromosome localization was deemed “abnormal” if the ratio of the shortest chromosome-cortex distance at anaphase initiation (DA) to that at GVBD (DGV) was greater than 0.6 (DA/DGV >0.6). Furrow localization was deemed “abnormal” if DL/DS was less than 1.9 (DL/DS <1.9). (TIF) [file pone.0029735.s002.tif]

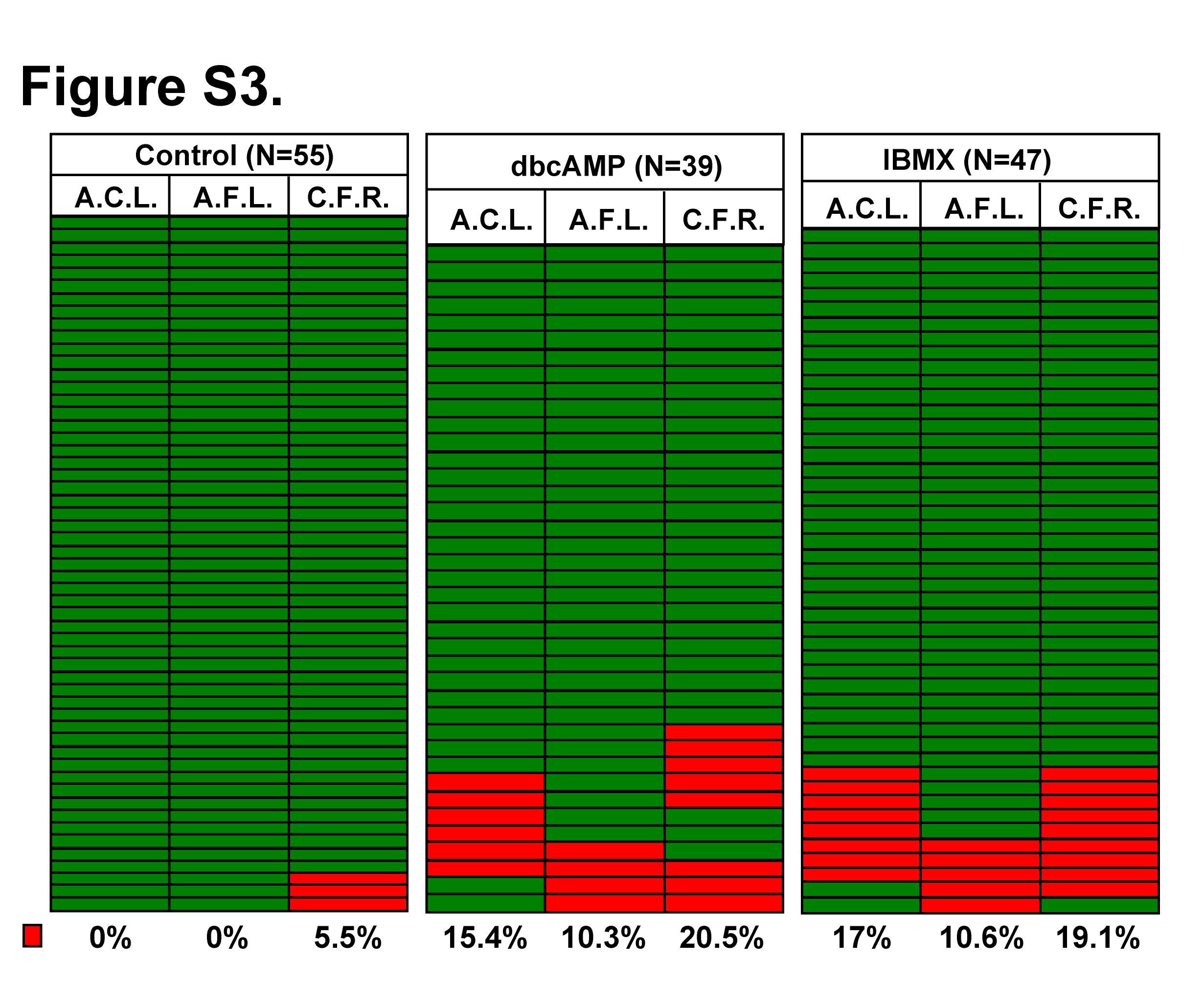

Supplement: Figure S3 — Incubation with 0.2 mM IBMX or 0.3 mM dbcAMP did not significantly affect post-GVBD oocytes. Denuded mouse oocytes at the GV stage were collected and cultured in M16 medium for 90 min; then, oocytes that had undergone GVBD were transferred into DMSO control medium or medium supplemented with 0.3 mM dbcAMP or 0.2 mM IBMX for live-cell imaging. Shown is a summary of normal and abnormal cytokinesis in control, dbcAMP and IBMX groups, with each row representing a single oocyte division. The cell divisions are indicated in different colors: presence (red) or absence (green) of abnormal anaphase chromosome localization (A.C.L., first column) or abnormal cleavage furrow localization (A.F.L., second column); occurrence of cleavage furrow regression (red) or not (green) (C.F.R., third column). The proportion of red in each column is included below. Chromosome localization was deemed “abnormal” when DA/DO >0.68. The vertical distances from the far-end cortex to the cleavage furrow in larger and smaller daughter cells were measured in each oocyte, and furrow localization was deemed “abnormal” if DL/DS <1.70. (TIF) [file pone.0029735.s003.tif]

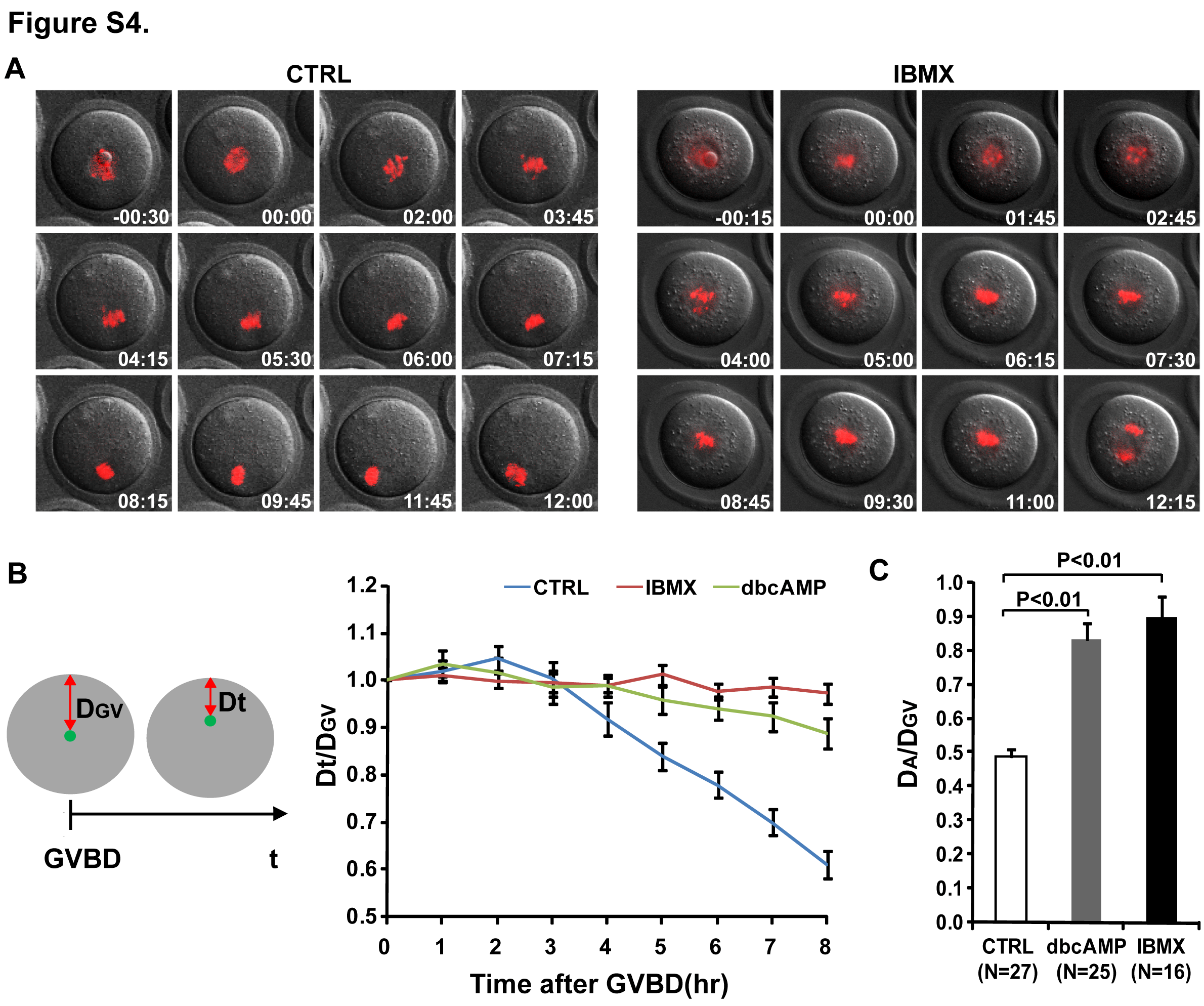

Supplement: Figure S4 — IBMX and dbcAMP treatment on oocytes at GV stage disturbed chromosome migration. Chromosome movement was tracked by live cell imaging in control oocytes, and the oocytes treated for 20 hours with 0.2 mM IBMX or 0.3 mM dbcAMP. (A) Representative time lapse images of chromosome movement from GVBD to anaphase onset. The time of GVBD was set as 00∶00 (hours∶minutes). Red: DNA. (B) Green dots indicate the position of the chromosome cluster. The shortest distance between the chromosomes and the cortex was measured at each time point (Dt) and divided by the distance to the cortex at GVBD (DGV). Dt/DGV plotted against time was used as an indicator of chromosome movement. Panel (C) shows the relative position of the chromosome cluster (i.e., average DA/DGV) at anaphase onset. DA is the shortest distance between the chromosomes and the cortex at anaphase initiation. P values are from a t-test. (TIF) [file pone.0029735.s004.tif]

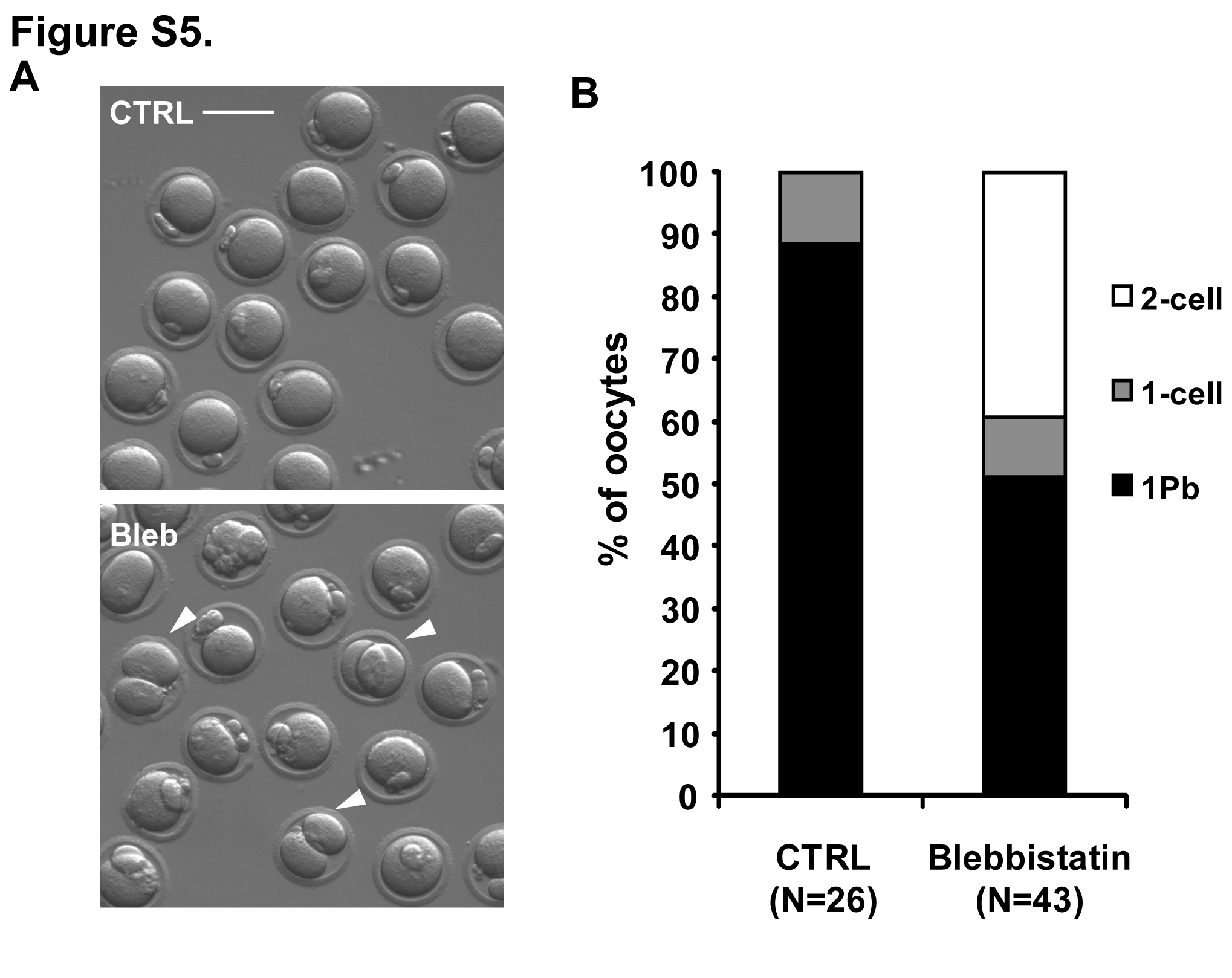

Supplement: Figure S5 — Blebbistatin induces symmetric meiosis I division in mouse oocytes. (A) Oocytes were incubated with (Bleb) or without (CTRL) 0.2 mM blebbistatin for 16 hours. Arrowheads indicate oocytes that produced 2 daughter cells of similar size (“2-cells”). (B) The frequency of 2-cells was elevated by the presence of 0.2 mM blebbistatin. (TIF) [file pone.0029735.s005.tif]
